# The genome sequence of an anthomyiid fly, *Pegoplata infirma *(Meigen, 1826) (Diptera, Anthomyiidae)

**DOI:** 10.12688/wellcomeopenres.22463.1

**Published:** 2024-06-21

**Authors:** Steven Falk, Philip Brighton, University of Oxford and Wytham Woods Genome Acquisition Lab, Darwin Tree of Life Barcoding collective, Wellcome Sanger Institute Tree of Life Management, Samples and Laboratory team, Wellcome Sanger Institute Scientific Operations: Sequencing Operations, Wellcome Sanger Institute Tree of Life Core Informatics team, Tree of Life Core Informatics collective, Darwin Tree of Life Consortium

**Affiliations:** 1 Independent researcher, Kenilworth, England, UK; 2 Independent researcher, Organiser of UK Anthomyiidae Recording Scheme for the Biological Records Centre, Warrington, England, UK

**Keywords:** Pegoplata infirma, anthomyiid fly, genome sequence, chromosomal, Diptera

## Abstract

We present a genome assembly from an individual male *Pegoplata infirma* (anthomyiid fly; Arthropoda; Insecta; Diptera; Anthomyiidae). The genome sequence is 1384.4 megabases in span. Most of the assembly is scaffolded into 7 chromosomal pseudomolecules, including the X and Y sex chromosomes. The mitochondrial genome has also been assembled and is 16.15 kilobases in length.

## Species taxonomy

Eukaryota; Opisthokonta; Metazoa; Eumetazoa; Bilateria; Protostomia; Ecdysozoa; Panarthropoda; Arthropoda; Mandibulata; Pancrustacea; Hexapoda; Insecta; Dicondylia; Pterygota; Neoptera; Endopterygota; Diptera; Brachycera; Muscomorpha; Eremoneura; Cyclorrhapha; Schizophora; Calyptratae; Muscoidea; Anthomyiidae; Pegomyinae; *Pegoplata*; *Pegoplata infirma* (Meigen, 1826) (NCBI:txid559701).

## Background

British species of the genus *Pegoplata* are typical dark grey or blackish bristly calyptrate flies. The rear tibiae have just two anterodorsal and two posterodorsal setae in matched pairs; the sternite V is heart-shaped; and the palps are usually dilated in females. Further details for identification of *P. infirma*, including diagrams of the male genitalia, are given by Ackland *et al.* (2017).

*Pegoplata infirma* was historically classified under the genus *Nupedia* by Hennig in 1976, alongside five other species ( Hennig, 1976). All of these, bar one ( *tundrica* Schnabl), have been recorded in Britain ( Chandler, 2023). *Nupedia* was subsequently merged with the genus *Pegoplata* Schnabl & Dziedzicki. The Global Biodiversity Information Facility currently lists 41 species in this genus ( GBIF Secretariat, 2024a). Current classifications, including recent genetic studies, place *Pegoplata infirma* firmly within the tribe Myopini of the subfamily Pegomyinae ( Chandler, 2023). The classification of Li *et al.* (2023), based on mitochondrial genomes, including that of *Pegoplata infirma*, is largely in agreement with this.

*Pegoplata infirma* is distributed through northern Europe and Scandinavia with a swathe across the middle latitudes of North America ( GBIF Secretariat, 2024b). In addition, it is documented from Iceland ( Ólafsson & Ingimarsdóttir, 2009), Japan, China and Kamchatka ( Suwa, 1999). Data from the National Biodiversity Network ( NBN Partnership, 2024) show *Pegoplata infirma* distributed across the whole of Britain including outlying islands. It is amongst the five most frequently recorded of the 246 British anthomyiid species ( Ackland *et al.*, 2017).

The lifecycle and breeding habits of *Pegoplata infirma* are closely linked to specific environmental conditions, particularly within livestock-related habitats. Skidmore (1991) lists *Nupedia* larvae amongst the British fauna of cow-dung, and Griffiths (1997) states that saprophagous larvae feeding on dung or other decomposing material are found in several genera including *Pegoplata.* In an extensive compendium of Diptera associations, Skidmore (2010) lists *Pegoplata infirma* as breeding only in sheep dung. Chandler (2021) reports an adult at primrose flowers and another on cow dung. Ólafsson and Ingimarsdóttir (2009) list *Pegoplata infirma* as “settled” on the volcanic island of Surtsey, suggesting that seabird dung or other detritus is also on the menu. However, Wolton and Field (2021) found only one specimen of *Pegoplata infirma* amongst 4,105 individual Diptera captured in emergence traps in a cattle-grazed wet woodland. These and other records ( Barták, 1998; Brighton, 2020, Brighton, 2022; Skidmore, 2006) indicate an absence of any specific habitat preferences, and a wide tolerance of environmental conditions.

It appears that while so frequently and so widely encountered as an adult, *Pegoplata infirma* is a relatively minor component of the dung community. Adults can be distinguished from other small Anthomyiid and Muscid flies only by capture and microscopic examination, limiting the scope for observations of adult behaviour. There is thus great scope for new genome data which could shed light on the biology of the species.

## Genome sequence report

**Figure 1.**
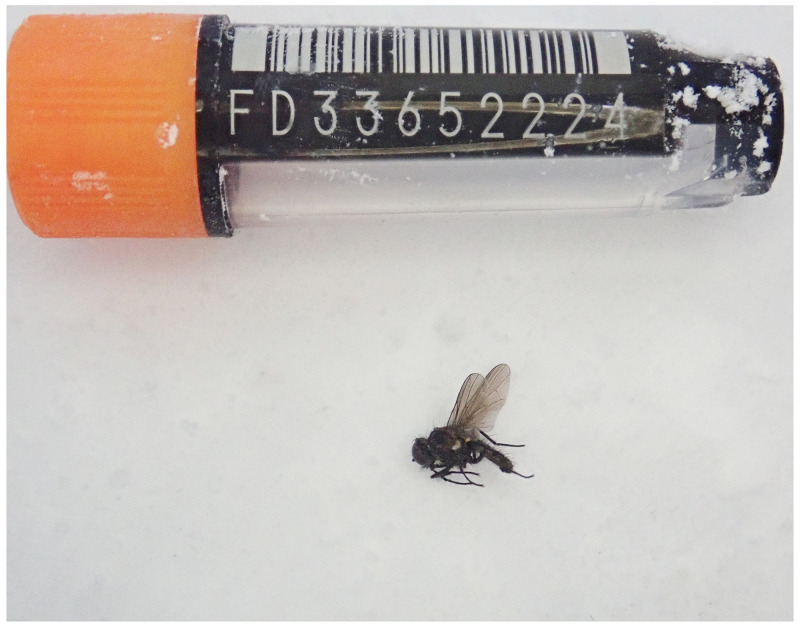
Photograph of the *Pegoplata infirma* (idPegInfi2) specimen used for genome sequencing.

The genome was sequenced from adult male *Pegoplata infirma* ( Figure 1) collected from Wytham Woods, Oxfordshire, UK (51.77, –1.33). A total of 25-fold coverage in Pacific Biosciences single-molecule HiFi long reads was generated. Primary assembly contigs were scaffolded with chromosome conformation Hi-C data. Manual assembly curation corrected 243 missing joins or mis-joins and removed 114 haplotypic duplications, reducing the assembly length by 6.39%and the scaffold number by 7.32%, and increasing the scaffold N50 by 49.36%.

**Figure 2.**
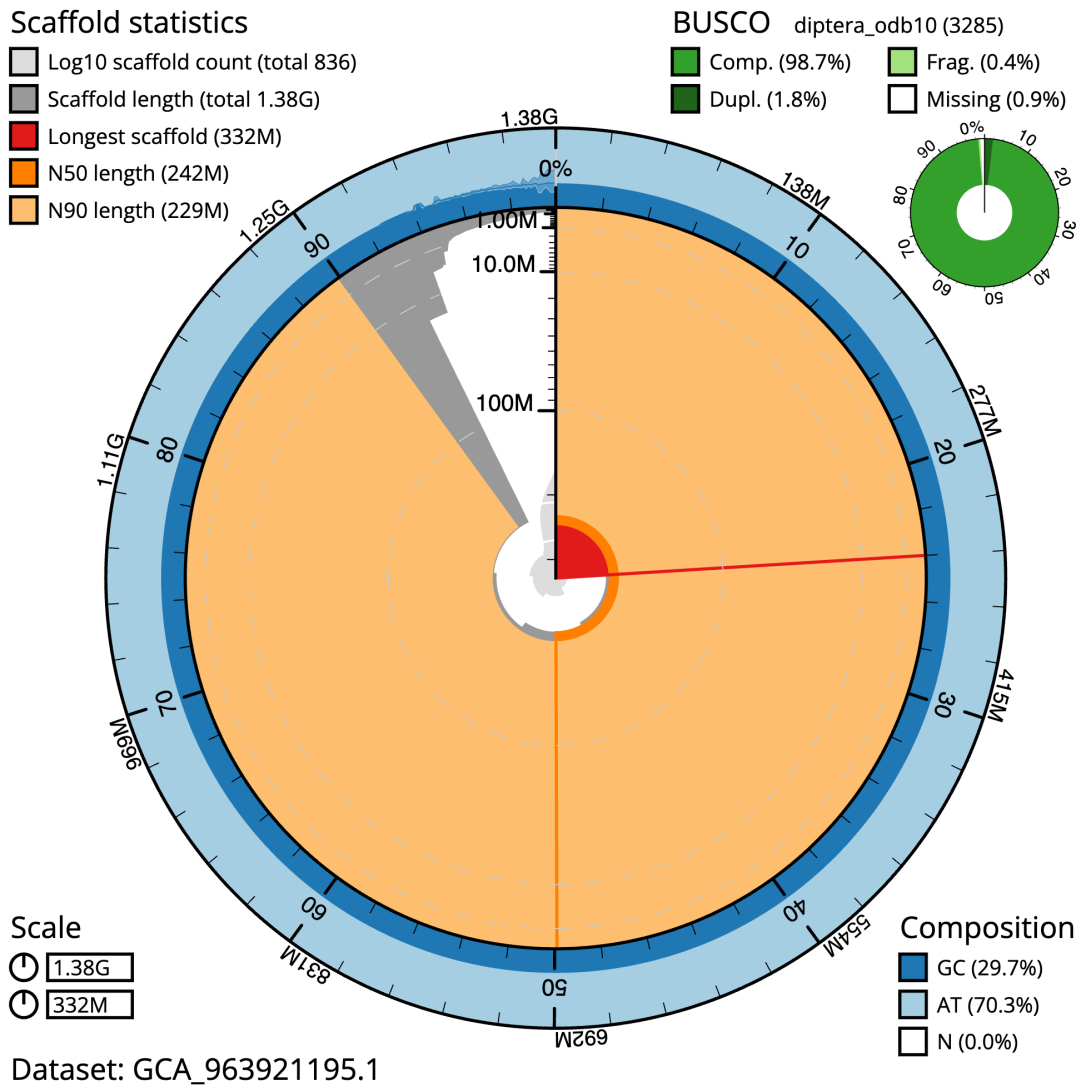
Genome assembly of *Pegoplata infirma*, idPegInfi2.1: metrics. The BlobToolKit snail plot shows N50 metrics and BUSCO gene completeness. The main plot is divided into 1,000 size-ordered bins around the circumference with each bin representing 0.1% of the 1,384,385,935 bp assembly. The distribution of scaffold lengths is shown in dark grey with the plot radius scaled to the longest scaffold present in the assembly (332,497,885 bp, shown in red). Orange and pale-orange arcs show the N50 and N90 scaffold lengths (241,705,591 and 228,884,631 bp), respectively. The pale grey spiral shows the cumulative scaffold count on a log scale with white scale lines showing successive orders of magnitude. The blue and pale-blue area around the outside of the plot shows the distribution of GC, AT and N percentages in the same bins as the inner plot. A summary of complete, fragmented, duplicated and missing BUSCO genes in the diptera_odb10 set is shown in the top right. An interactive version of this figure is available at https://blobtoolkit.genomehubs.org/view/Pegoplata_infirma/dataset/GCA_963921195.1/snail.

**Figure 3.**
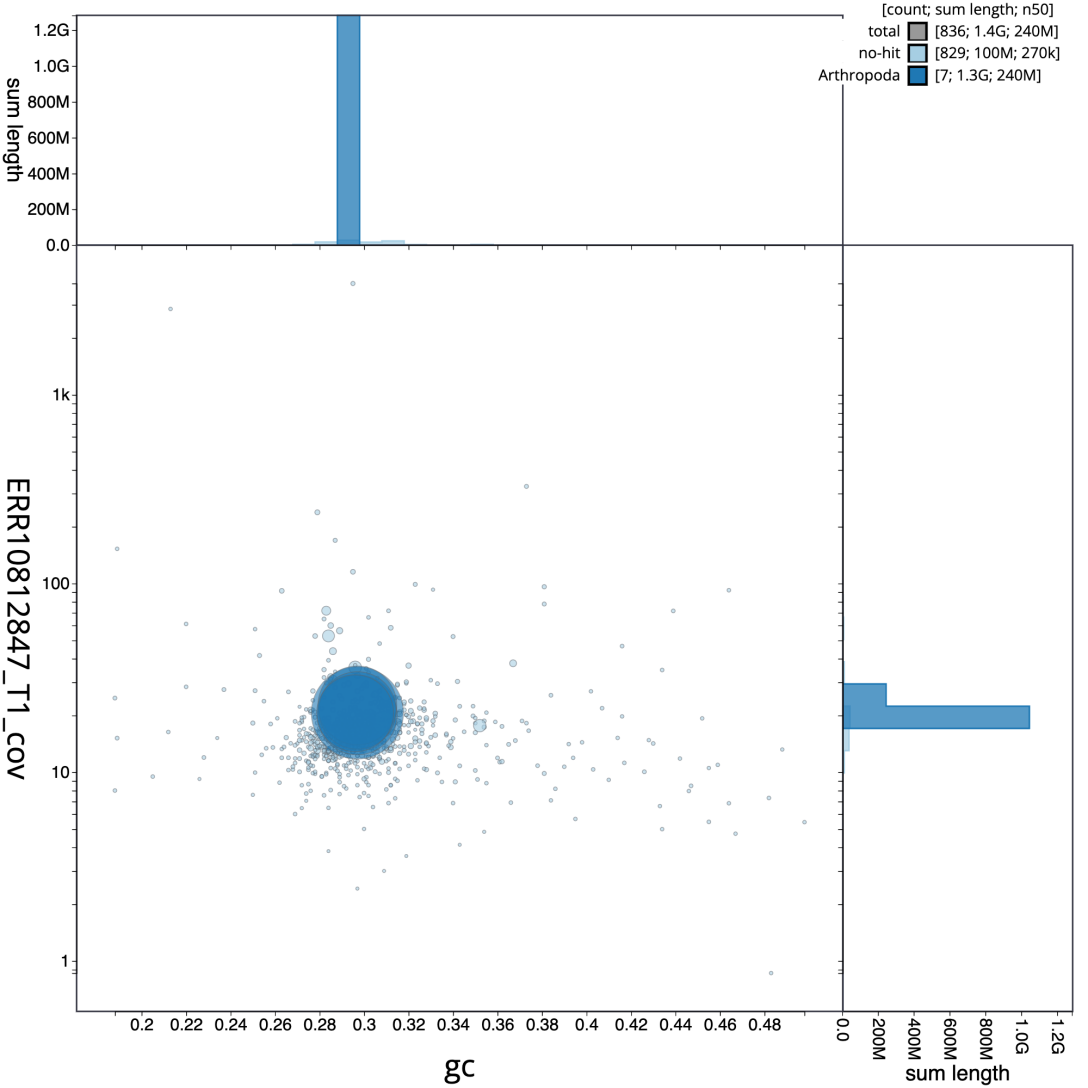
Genome assembly of *Pegoplata infirma*, idPegInfi2.1: BlobToolKit GC-coverage plot. Sequences are coloured by phylum. Circles are sized in proportion to sequence length. Histograms show the distribution of sequence length sum along each axis. An interactive version of this figure is available at https://blobtoolkit.genomehubs.org/view/Pegoplata_infirma/dataset/GCA_963921195.1/blob.

**Figure 4.**
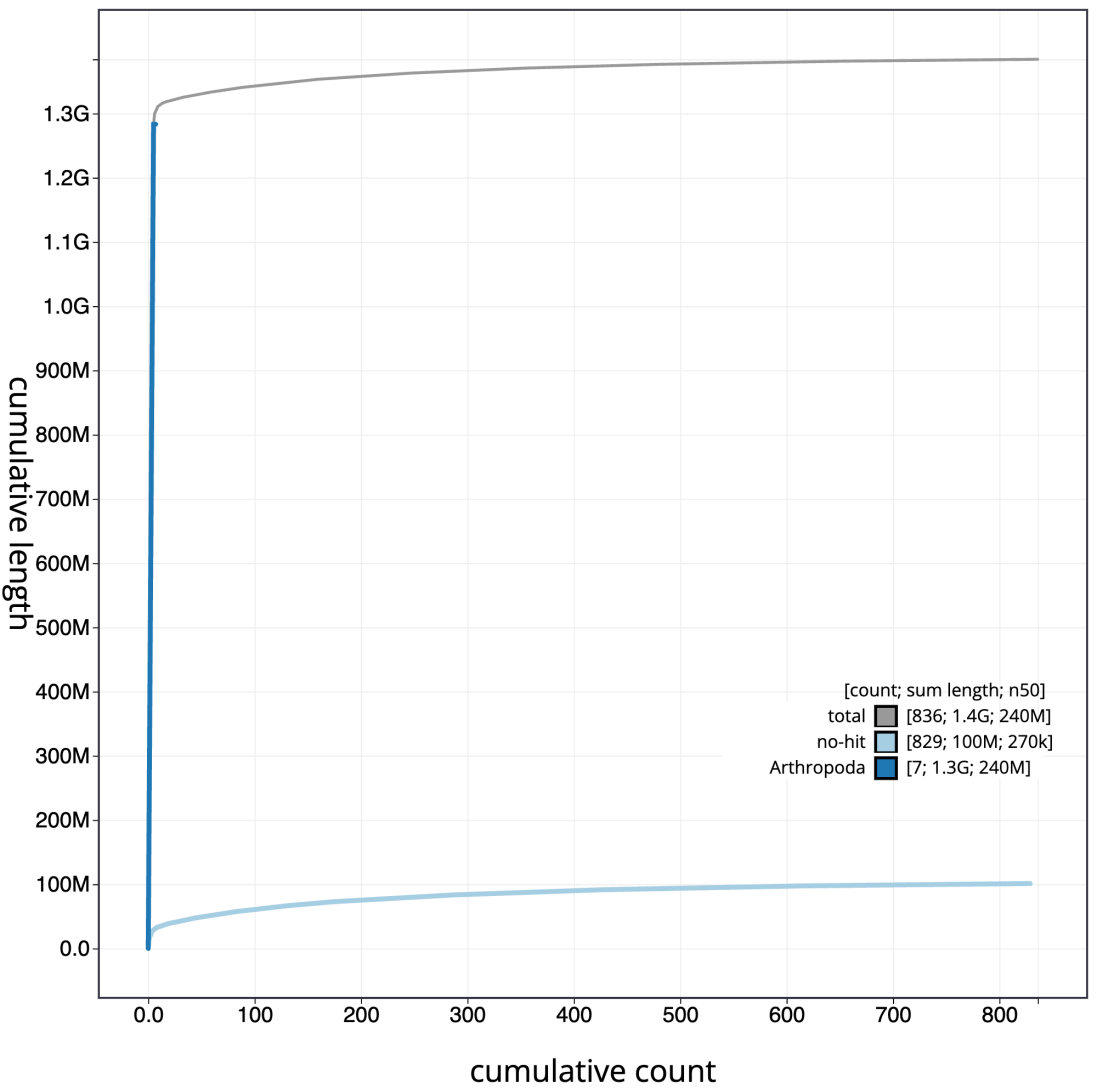
Genome assembly of *Pegoplata infirma* idPegInfi2.1: BlobToolKit cumulative sequence plot. The grey line shows cumulative length for all sequences. Coloured lines show cumulative lengths of sequences assigned to each phylum using the buscogenes taxrule. An interactive version of this figure is available at https://blobtoolkit.genomehubs.org/view/Pegoplata_infirma/dataset/GCA_963921195.1/cumulative.

**Figure 5.**
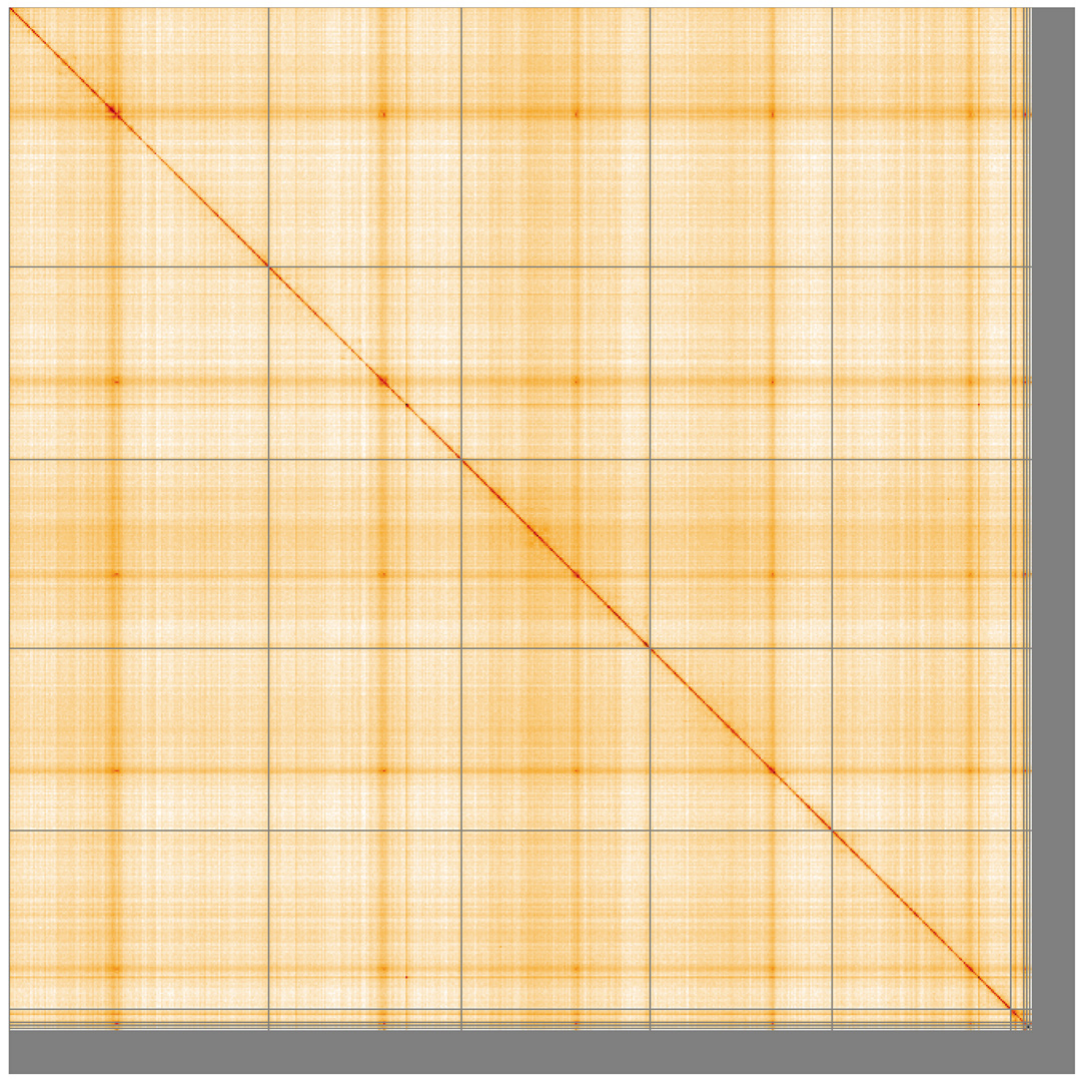
Genome assembly of *Pegoplata infirma* idPegInfi2.1: Hi-C contact map of the idPegInfi2.1 assembly, visualised using HiGlass. Chromosomes are shown in order of size from left to right and top to bottom. An interactive version of this figure may be viewed at https://genome-note-higlass.tol.sanger.ac.uk/l/?d=BXbq1MfOQk6D13Hi_pheug.

The final assembly has a total length of 1,384.4 Mb in 835 sequence scaffolds with a scaffold N50 of 241.7 Mb ( Table 1). The snail plot in Figure 2 provides a summary of the assembly statistics, while the distribution of assembly scaffolds on GC proportion and coverage is shown in Figure 3. The cumulative assembly plot in Figure 4 shows curves for subsets of scaffolds assigned to different phyla. Most (94.15%) of the assembly sequence was assigned to 7 chromosomal-level scaffolds, representing 5 autosomes and the X and Y sex chromosomes. Chromosome-scale scaffolds confirmed by the Hi-C data are named in order of size ( Figure 5; Table 2). Chromosomes X and Y were assigned based on read coverage statistics. While not fully phased, the assembly deposited is of one haplotype. Contigs corresponding to the second haplotype have also been deposited. The mitochondrial genome was also assembled and can be found as a contig within the multifasta file of the genome submission.

**Table 1.** Genome data for *Pegoplata infirma*, idPegInfi2.1.

Project accession data		
Assembly identifier	idPegInfi2.1	
Species	*Pegoplata infirma*	
Specimen	idPegInfi2	
NCBI taxonomy ID	559701	
BioProject	PRJEB59157	
BioSample ID	Genome: SAMEA110451833 Hi-C:	
Isolate information	idPegInfi2: whole organism (genome sequence) idPegInfi1: whole organism (Hi-C sequencing)	
Assembly metrics *		*Benchmark*
Consensus quality (QV)	61.3	*≥ 50*
*k*-mer completeness	100.0%	*≥ 95%*
BUSCO **	C:98.7%[S:96.9%,D:1.8%], F:0.5%,M:0.8%,n:3,285	*C ≥ 95%*
Percentage of assembly mapped to chromosomes	94.15%	*≥ 95%*
Sex chromosomes	XY	*localised homologous pairs*
Organelles	Mitochondrial genome: 16.15 kb	*complete single alleles*
Raw data accessions		
PacificBiosciences Sequel IIe	ERR10812847	
Hi-C Illumina	ERR10802479	
Genome assembly		
Assembly accession	GCA_963921195.1	
*Accession of alternate haplotype*	GCA_963921205.1	
Span (Mb)	1384.4	
Number of contigs	2052	
Contig N50 length (Mb)	2.3	
Number of scaffolds	835	
Scaffold N50 length (Mb)	241.7	
Longest scaffold (Mb)	332.5	

* Assembly metric benchmarks are adapted from column VGP-2020 of “Table 1: Proposed standards and metrics for defining genome assembly quality” from Rhie *et al.* (2021). ** BUSCO scores based on the diptera_odb10 BUSCO set using version 5.4.3. C = complete [S = single copy, D = duplicated], F = fragmented, M = missing, n = number of orthologues in comparison. A full set of BUSCO scores is available at https://blobtoolkit.genomehubs.org/view/Pegoplata_infirma/dataset/GCA_963921195.1/busco.

**Table 2.** Chromosomal pseudomolecules in the genome assembly of *Pegoplata infirma*, idPegInfi2.

INSDC accession	Chromosome	Length (Mb)	GC%
OY992536.1	1	332.5	29.5
OY992537.1	2	246.88	29.5
OY992538.1	3	241.71	29.5
OY992539.1	4	233.04	29.5
OY992540.1	5	228.88	29.5
OY992541.1	X	16.69	31.0
OY992542.1	Y	3.78	35.0
OY992543.1	MT	0.02	21.5

The estimated Quality Value (QV) of the final assembly is 61.3 with *k*-mer completeness of 100.0%, and the assembly has a BUSCO v5.4.3 completeness of 98.7% (single = 96.9%, duplicated = 1.8%), using the diptera_odb10 reference set ( *n* = 3,285).

Metadata for specimens, barcode results, spectra estimates, sequencing runs, contaminants and pre-curation assembly statistics are given at https://links.tol.sanger.ac.uk/species/559701.

## Methods

### Sample acquisition and nucleic acid extraction

A male adult *Pegoplata infirma* (specimen ID Ox002182, ToLID idPegInfi2) was collected from Wytham Woods, Oxfordshire, UK (latitude 51.77, longitude –1.33) on 2022-05-19 by netting. The specimen used for Hi-C sequencing (specimen ID Ox000905, ToLID idPegInfi1) was an adult specimen netted in the same location on 2020-08-03. Both specimens were collected and identified by Steven Falk (independent researcher), and preserved on dry ice.

The workflow for high molecular weight (HMW) DNA extraction at the Wellcome Sanger Institute (WSI) Tree of Life Core Laboratory includes a sequence of core procedures: sample preparation; sample homogenisation, DNA extraction, fragmentation, and clean-up. In sample preparation, the idPegInfi2 sample was weighed and dissected on dry ice ( Jay *et al.*, 2023).

Tissue from the whole organism was homogenised using a PowerMasher II tissue disruptor ( Denton *et al.*, 2023a). HMW DNA was extracted using the Automated MagAttract v1 protocol ( Sheerin *et al.*, 2023). DNA was sheared into an average fragment size of 12–20 kb in a Megaruptor 3 system with speed setting 30 ( Todorovic *et al.*, 2023). Sheared DNA was purified by solid-phase reversible immobilisation ( Strickland *et al.*, 2023): in brief, the method employs a 1.8X ratio of AMPure PB beads to sample to eliminate shorter fragments and concentrate the DNA. The concentration of the sheared and purified DNA was assessed using a Nanodrop spectrophotometer and Qubit Fluorometer and Qubit dsDNA High Sensitivity Assay kit. Fragment size distribution was evaluated by running the sample on the FemtoPulse system.

Protocols developed by the WSI Tree of Life laboratory are publicly available on protocols.io ( Denton *et al.*, 2023b).

### Sequencing

Pacific Biosciences HiFi circular consensus DNA sequencing libraries were constructed according to the manufacturers’ instructions. DNA sequencing was performed by the Scientific Operations core at the WSI on a Pacific Biosciences Sequel IIe (HiFi) instrument. Hi-C data were also generated from whole organism tissue of idPegInfi1 using the Arima v2 kit. The Hi-C sequencing was performed using paired-end sequencing with a read length of 150 bp on the Illumina NovaSeq 6000 instrument.

### Genome assembly and curation

Assembly was carried out with Hifiasm ( Cheng *et al.*, 2021) and haplotypic duplication was identified and removed with purge_dups ( Guan *et al.*, 2020). The assembly was then scaffolded with Hi-C data ( Rao *et al.*, 2014) using YaHS ( Zhou *et al.*, 2023). The assembly was checked for contamination and corrected using the TreeVal pipeline ( Pointon *et al.*, 2023). Manual curation was performed using JBrowse2 ( Diesh *et al.*, 2023), HiGlass ( Kerpedjiev *et al.*, 2018) and PretextView ( Harry, 2022). The mitochondrial genome was assembled using MitoHiFi ( Uliano-Silva *et al.*, 2023), which runs MitoFinder ( Allio *et al.*, 2020) or MITOS ( Bernt *et al.*, 2013) and uses these annotations to select the final mitochondrial contig and to ensure the general quality of the sequence.

### Evaluation of final assembly

The final assembly was post-processed and evaluated with the three Nextflow ( Di Tommaso *et al.*, 2017) DSL2 pipelines “sanger-tol/readmapping” ( Surana *et al.*, 2023a), “sanger-tol/genomenote” ( Surana *et al.*, 2023b), and “sanger-tol/blobtoolkit” ( Muffato *et al.*, 2024). The pipeline sanger-tol/readmapping aligns the Hi-C reads with bwa-mem2 ( Vasimuddin *et al.*, 2019) and combines the alignment files with SAMtools ( Danecek *et al.*, 2021). The sanger-tol/genomenote pipeline transforms the Hi-C alignments into a contact map with BEDTools ( Quinlan & Hall, 2010) and the Cooler tool suite ( Abdennur & Mirny, 2020), which is then visualised with HiGlass ( Kerpedjiev *et al.*, 2018). It also provides statistics about the assembly with the NCBI datasets ( Sayers *et al.*, 2024) report, computes *k*-mer completeness and QV consensus quality values with FastK and MerquryFK, and a completeness assessment with BUSCO ( Manni *et al.*, 2021).

The sanger-tol/blobtoolkit pipeline is a Nextflow port of the previous Snakemake Blobtoolkit pipeline ( Challis *et al.*, 2020). It aligns the PacBio reads with SAMtools and minimap2 ( Li, 2018) and generates coverage tracks for regions of fixed size. In parallel, it queries the GoaT database ( Challis *et al.*, 2023) to identify all matching BUSCO lineages to run BUSCO ( Manni *et al.*, 2021). For the three domain-level BUSCO lineage, the pipeline aligns the BUSCO genes to the Uniprot Reference Proteomes database ( Bateman *et al.*, 2023) with DIAMOND ( Buchfink *et al.*, 2021) blastp. The genome is also split into chunks according to the density of the BUSCO genes from the closest taxonomically lineage, and each chunk is aligned to the Uniprot Reference Proteomes database with DIAMOND blastx. Genome sequences that have no hit are then chunked with seqtk and aligned to the NT database with blastn ( Altschul *et al.*, 1990). All those outputs are combined with the blobtools suite into a blobdir for visualisation.

All three pipelines were developed using the nf-core tooling ( Ewels *et al.*, 2020), use MultiQC ( Ewels *et al.*, 2016), and make extensive use of the Conda package manager, the Bioconda initiative ( Grüning *et al.*, 2018), the Biocontainers infrastructure ( da Veiga Leprevost *et al.*, 2017), and the Docker ( Merkel, 2014) and Singularity ( Kurtzer *et al.*, 2017) containerisation solutions.

Table 3 contains a list of relevant software tool versions and sources.

**Table 3.** Software tools: versions and sources.

Software tool	Version	Source
BEDTools	2.30.0	https://github.com/arq5x/bedtools2
Blast	2.14.0	ftp://ftp.ncbi.nlm.nih.gov/blast/executables/blast+/
BlobToolKit	4.3.7	https://github.com/blobtoolkit/blobtoolkit
BUSCO	5.4.3 and 5.5.0	https://gitlab.com/ezlab/busco
bwa-mem2	2.2.1	https://github.com/bwa-mem2/bwa-mem2
Cooler	0.8.11	https://github.com/open2c/cooler
DIAMOND	2.1.8	https://github.com/bbuchfink/diamond
fasta_windows	0.2.4	https://github.com/tolkit/fasta_windows
FastK	427104ea91c78c3b8b8b49f1a7d6bbeaa869ba1c	https://github.com/thegenemyers/FASTK
GoaT CLI	0.2.5	https://github.com/genomehubs/goat-cli
Hifiasm	0.16.1-r375	https://github.com/chhylp123/hifiasm
HiGlass	44086069ee7d4d3f6f3f0012569789ec138f42b84aa44357826c0b6753eb28de	https://github.com/higlass/higlass
MerquryFK	d00d98157618f4e8d1a9190026b19b471055b22e	https://github.com/thegenemyers/MERQURY.FK
MitoHiFi	2	https://github.com/marcelauliano/MitoHiFi
MultiQC	1.14, 1.17, and 1.18	https://github.com/MultiQC/MultiQC
NCBI Datasets	15.12.0	https://github.com/ncbi/datasets
Nextflow	23.04.0-5857	https://github.com/nextflow-io/nextflow
PretextView	0.2	https://github.com/wtsi-hpag/PretextView
purge_dups	1.2.3	https://github.com/dfguan/purge_dups
samtools	1.16.1, 1.17, and 1.18	https://github.com/samtools/samtools
sanger-tol/genomenote	1.1.1	https://github.com/sanger-tol/genomenote
sanger-tol/readmapping	1.2.1	https://github.com/sanger-tol/readmapping
Seqtk	1.3	https://github.com/lh3/seqtk
Singularity	3.9.0	https://github.com/sylabs/singularity
TreeVal	1.0.0	https://github.com/sanger-tol/treeval
YaHS	yahs-1.1.91eebc2	https://github.com/c-zhou/yahs

### Wellcome Sanger Institute – Legal and Governance

The materials that have contributed to this genome note have been supplied by a Darwin Tree of Life Partner. The submission of materials by a Darwin Tree of Life Partner is subject to the **‘Darwin Tree of Life Project Sampling Code of Practice’**, which can be found in full on the Darwin Tree of Life website here. By agreeing with and signing up to the Sampling Code of Practice, the Darwin Tree of Life Partner agrees they will meet the legal and ethical requirements and standards set out within this document in respect of all samples acquired for, and supplied to, the Darwin Tree of Life Project.

Further, the Wellcome Sanger Institute employs a process whereby due diligence is carried out proportionate to the nature of the materials themselves, and the circumstances under which they have been/are to be collected and provided for use. The purpose of this is to address and mitigate any potential legal and/or ethical implications of receipt and use of the materials as part of the research project, and to ensure that in doing so we align with best practice wherever possible. The overarching areas of consideration are:

•      Ethical review of provenance and sourcing of the material

•      Legality of collection, transfer and use (national and international)

Each transfer of samples is further undertaken according to a Research Collaboration Agreement or Material Transfer Agreement entered into by the Darwin Tree of Life Partner, Genome Research Limited (operating as the Wellcome Sanger Institute), and in some circumstances other Darwin Tree of Life collaborators.

## Data Availability

European Nucleotide Archive: *Pegoplata infirma*. Accession number PRJEB59157; https://identifiers.org/ena.embl/PRJEB59157 ( Wellcome Sanger Institute, 2024). The genome sequence is released openly for reuse. The *Pegoplata infirma* genome sequencing initiative is part of the Darwin Tree of Life (DToL) project. All raw sequence data and the assembly have been deposited in INSDC databases. The genome will be annotated using available RNA-Seq data and presented through the Ensembl pipeline at the European Bioinformatics Institute. Raw data and assembly accession identifiers are reported in Table 1.

## Author information

Members of the University of Oxford and Wytham Woods Genome Acquisition Lab are listed here: https://doi.org/10.5281/zenodo.7125292.

Members of the Darwin Tree of Life Barcoding collective are listed here: https://doi.org/10.5281/zenodo.4893703.

Members of the Wellcome Sanger Institute Tree of Life Management, Samples and Laboratory team are listed here: https://doi.org/10.5281/zenodo.10066175.

Members of Wellcome Sanger Institute Scientific Operations: Sequencing Operations are listed here: https://doi.org/10.5281/zenodo.10043364.

Members of the Wellcome Sanger Institute Tree of Life Core Informatics team are listed here: https://doi.org/10.5281/zenodo.10066637.

Members of the Tree of Life Core Informatics collective are listed here: https://doi.org/10.5281/zenodo.5013541.

Members of the Darwin Tree of Life Consortium are listed here: https://doi.org/10.5281/zenodo.4783558.
