# Supplementary material for: The genome sequence of an anthomyiid fly, Pegoplata infirma (Meigen, 1826) (Diptera, Anthomyiidae)
Source: Wellcome Open Res. 2024 Jun 21;9:330. [Version 1] doi: 10.12688/wellcomeopenres.22463.1 (PMC11937944; doi:10.12688/wellcomeopenres.22463.1)
Supplement: Peer review history

# Reviewer report: Reviewer response for version 1

**DOI:** 10.21956/wellcomeopenres.24751.r98447

**Published:** 2024-09-12

**Authors:** Marcus Stensmyr

**Affiliations:** 1 Lund University, Lund, Sweden

In the submitted manuscript, Falk and Brighton present a chromosome-level genome assembly for the dung-feeding anthomyiid fly, Pegoplata infirma. The assembly is of high quality, encompassing 1384.4 megabases assembled into 7 scaffolds of chromosome length (including two putative sex chromosomes), as well as a mitochondrial genome. The authors acknowledge the absence of preliminary annotations, which may limit its immediate utility for further research. That said, annotation will be done via the Ensemble pipeline in the future. The methods employed for sequencing as well as assembly were conducted in accordance with established procedures. Overall, the work contributes valuable genomic data from an interesting group of flies.

Are sufficient details of methods and materials provided to allow replication by others?

Yes

Is the rationale for creating the dataset(s) clearly described?

Yes

Are the datasets clearly presented in a useable and accessible format?

Yes

Are the protocols appropriate and is the work technically sound?

Yes

Reviewer Expertise:

Insect neurogenetics and genomics

I confirm that I have read this submission and believe that I have an appropriate level of expertise to confirm that it is of an acceptable scientific standard.

# Reviewer report: Reviewer response for version 1

**DOI:** 10.21956/wellcomeopenres.24751.r93025

**Published:** 2024-08-27

**Authors:** Kyle M Benowitz

**Affiliations:** 1 College of Integrative Sciences and Arts, Arizona State University - Polytechnic Campus (Ringgold ID: 52448), Mesa, Arizona, USA

The note presents a chromosome level genome assembly for a dung-feeding anthomyiid fly. The assembly is of high quality, but unfortunately there is not even a preliminary annotation provided, which will limit its usefulness to other researchers for the moment. I have a few minor comments but otherwise I'm glad to see this genome published.

 1. I would like to see more justification on why this species was chosen for genome assembly. The authors provide basic detail on the morphology, phylogenetic position, and natural history of the organism. But I don't see any indication of why the genome was sequenced except a vague statement that it "could shed light on the biology of the species." The authors should consider adding explicit statements on the biological questions that can be addressed using this genome.

 2. In the methods, could the authors elaborate on how the fly was caught via "netting"? Without a bit more detail on the methods for collecting, I'm not sure the work is replicable.

Are sufficient details of methods and materials provided to allow replication by others?

Partly

Is the rationale for creating the dataset(s) clearly described?

Partly

Are the datasets clearly presented in a useable and accessible format?

Yes

Are the protocols appropriate and is the work technically sound?

Yes

Reviewer Expertise:

Insect evolutionary genomics

I confirm that I have read this submission and believe that I have an appropriate level of expertise to confirm that it is of an acceptable scientific standard.
